# Supplementary material for: Improving Drug Sensitivity of HIV-1 Protease Inhibitors by Restriction of Cellular Efflux System in a Fission Yeast Model
Source: Pathogens. 2022 Jul 16;11(7):804. doi: 10.3390/pathogens11070804 (PMC9318301; doi:10.3390/pathogens11070804)
Supplement: Supplementary file 1 [file pathogens-11-00804-s001.zip › ZhangJT22Pathogens_Table S1.pdf]

Table S1. Primers used in this study.

Name	Oligonucleotide sequence
Ble1-PR-F	AGATCTGTTTAGCTTGCCTCGTCCCCGCCGGGTCAC
Ble1-PR-R	GTTAACTGCGGTCAAGATATTTCTTGAATCAGG
AmpR-ori-F	GCTAGCATTATGGTGCACTCTCAGTACAATC
AmpR-ori-R	GCTAGCCTGCATTAATGAATCGGCCAACGCG
ura4-us-F	CGCGTTGGCCGATTCATTAATGCAGGCTAGCGATTGGAACTTTTGTTTTGCCCAAGG
ura4-us-R	CCTGATTCAAGAAATATCTTGACCGCAGTTAACCATGTGCAGAGATGCCGACGAAGC
ura4-ds-F	GTGACCCGGCGGGGACGAGGCAAGCTAAACAGATCTTCGATGCCTTGTTTGCGTTTGTT
ura4-ds-R	AGATTGTACTGAGAGTGCACCATAATGCTAGCCAGCTTGGCATTGTTCATACAAACG
PR-F	GTTAACTGCGGTCAAGATATTTCTTGAATCAGG
PR-R	CTGTCGCGGCCGCTTAAAAATTTAAAGTGCAGCCAA
ura4-FL-F	TGCCAGACCGTAATGACAAAACAGC
ura4-FL-R	GCGTAGTGTAGTATTGCTGACATTGGT

Note: F, forward primer; R, reverse primer.
