# Supplementary figures and images for: Improving Drug Sensitivity of HIV-1 Protease Inhibitors by Restriction of Cellular Efflux System in a Fission Yeast Model
Source: Pathogens. 2022 Jul 16;11(7):804. doi: 10.3390/pathogens11070804 (PMC9318301; doi:10.3390/pathogens11070804)

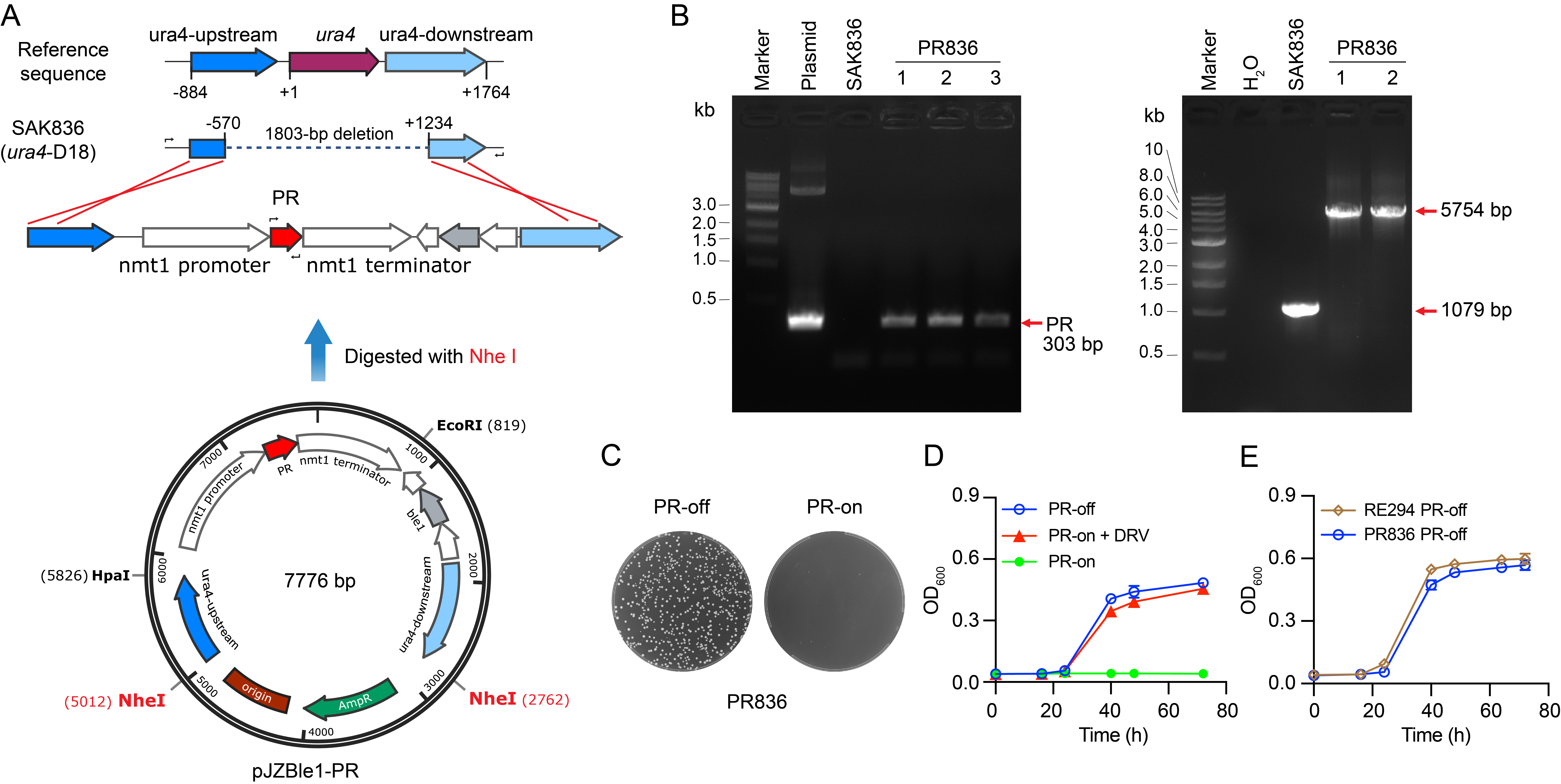

Supplement: Supplementary file 1 [file pathogens-11-00804-s001.zip › ZhangJT22Pathogens_Figure S1.jpg]
